# The Subjective Well-Being Challenge in the Accounting Profession: The Role of Job Resources

**DOI:** 10.3390/ijerph16173073

**Published:** 2019-08-23

**Authors:** Horacio Molina-Sánchez, Antonio Ariza-Montes, Mar Ortiz-Gómez, Antonio Leal-Rodríguez

**Affiliations:** 1 Department of Management, Universidad Loyola Andalucía, 14004 Córdoba, Spain; 2 Facultad de Administración y Negocios, Universidad Autónoma de Chile, Santiago 425, Chile; 3 Departamento de Administración de Empresas y Marketing, Universidad de Sevilla, 41018 Seville, Spain

**Keywords:** accountants, JDCS, auditor strains, subjective well-being

## Abstract

The main activity of the accountant is the preparation and audit of the financial information of a company. The subjective well-being of the accountant is important to ensure a balanced professional judgment and to offer a positive image of the profession in the face of the incorporation and retention of talent. However, accountants are subjected to intense pressures that affect their well-being in the performance of their tasks. In this paper, the job demands–resources theoretical framework is adopted to analyze the relationships between job demands, job resources, and the subjective well-being of a large sample of 739 accounting experts at the European level. Applying a structural equations model, the results confirm, on the one hand, the direct effects provided in the theoretical framework and, on the other, a new mediating role of job demands–subjective well-being relationship resources.

## 1. Introduction

At a societal level, subjective well-being must be considered a social objective of public health [1] due to its positive effects on longevity and the health of the employee [2].

Although subjective well-being is a heterogeneous and diffuse concept [2], in this paper, subjective well-being is formed with a construct with three dimensions: satisfaction with life, the presence of positive feelings and the absence of negative feelings, and flourishing [3]. Both subjective well-being and burnout, which reflects a deficient sense of well-being, have been conceptualized as mediators between the factors of job demands and performance or satisfaction with employment [4].

For instance, from the perspective of Job Demands Control Support model (JDCS) of [5,6], it can be spoken of up to four types of jobs: (1) Active Jobs would be those jobs with strong demands and a wide range of autonomy: These challenging jobs are common in certain professions, such as lawyers, journalists or managers, among others [7]. (2) Low-Strain Jobs are those jobs with low labor demands and high control over the activity, which can be translated into lower productivity. (3) High-Strain Jobs are characterized by intense physical and psychological demands, together with reduced margins of individual maneuverability regarding the level of autonomy and control. According to [7], roles which fall under this category would be machinery operators subjected to the rhythm of the machinery, office workers who perform automated and standardized tasks, or the telephone operators. (4) Finally, Passive Jobs are those with low labor demands and reduced control over the activity, which leads to routine and demotivation. Some examples of these types of jobs would be low-qualified service personnel: janitors, wardens, cleaners, etc.

All work is subject to some kind of pressure. Accountants have characteristics that make them particularly interesting as a collective who manage the combined effect of time pressures with conflict and role ambiguity [8,9,10,11]. In addition, few studies have investigated this group from the approach that is adopted in this paper, and that is addressed with a wide-ranging sample. Therefore, ensuring subjective well-being in a demanding profession that plays a relevant role in the economy is a research challenge. The theoretical framework of job demands–resources (JD-R) is one of the conceptual approaches used to explain relationships of the job with the subjective well-being of the employees [12,13]. This approach describes a relationship in which the job demands produce stress, while the resources available to the professional generate motivation.

The results should help managers decide whether improvement of subjective well-being would be based on the increase in the resources available or, conversely, in reducing the levels of pressure to which these professionals are subjected. On the one hand, an interactive effect would allow managers of these entities to consider maintaining current levels of demand because increasing available resources could mitigate the negative effect of the first on subjective well-being. In contrast, if the effect is additive in nature, the most effective way to improve levels of subjective well-being would be to reduce job demands [14].

Subjective well-being is evaluated in the JD-R framework through a double process, stress and motivation. Both influence outcome variables that are critical to the functioning of the organization (performance, turnover intentions, etc.) and for employee health. In fact, this approach is a framework that does not propose a single model, but it is a way of thinking that allows the implementation of various theories, which help to explain the relationships between job demands, resources, and well-being [15].

The direct relationship of job demands on stress and resources on motivation in various professions is widely contrasted (additive effects). However, the evidence of the effect of the interaction of job resources in the relationship between job demands and well-being is not as consistent [16,17,18].

### 1.1. Job Demands on Accountants

The accounting profession is subject to different demands, specific to this activity. The stressors on the auditors are classified into three types: role conflict, role ambiguity, and work overload [4]. Role conflict implies having to respond to two types of stressors that cannot be satisfied at the same time [19]; role ambiguity consists of a lack of information necessary to accomplish the task [20], and work overload is having too much work to perform on a tight deadline [21].

Empirical evidence shows that role conflict is high when judgments contain a higher burden of subjectivity [22], the position of professionals in the organization is lower [23], and the customer base is smaller [24]. Role conflict generates two types of stress, one from lack of information and control and the other from excessive work, which, in turn, have an impact on personal exhaustion, which is one of the dimensions of burnout [25].

In particular, auditors experienced pressure since they must be independent and skeptical and simultaneously maintain a good business relationship with the customer. Commercial pressure is especially intense at the beginning of a relationship, where the firm has made a commercial effort that it has to recover. At the personal level, accountants have an incentive to maintain a healthy client relationship because the financial departments of customers could be their next professional destination [26].

The uncertainty in the tasks to be carried out introduces role ambiguity; in fact, there is evidence that demonstrates that the auditors are more comfortable with more routine tasks than more complex tasks [23]. The ambiguity of role generates stress due to a lack of control and information that mediates the relationship with personal exhaustion.

The work of auditors and information preparers must be performed around the year-end. In the case of audit firms, there is also strong competition that requires adjusting budgets to be competitive that reduces implementation times; all this determines that the deadlines are very tight, such as with a high overload of work. Work overload is a stressor factor widely cited in the work of the professional accounting environment [27].

The effects that produce the demands on the performance or health are mediated by the production of tension. Additionally, the different demands have a direct effect on tension, or, in some works, it has been shown that the overload of work has a positive influence on the role conflict and role ambiguity [27].

The effects of job demands on job performance are an inverted U-shaped relationship, as it postulates the arousal theory [28,29,30,31,32] and explains the stress management in audit firms [33,34]. The JD-R model introduces the concept that this relationship is mediated by a variable of tension, e.g., burnout [35]. If the stressor fails to produce high levels of stress, pressure could be positive if it creates the effects of activation of resources; however, when the pressure causes stress, this translates into lower job performance. The negative influence of pressure on occupational health has been observed [9,36] and on job satisfaction [8].

Pressure also affects the quality of the work, indicating a negative relationship between stress factors and variables that denote dysfunctional behaviors by the auditors [37,38,39,40,41,42].

Several papers report that work overload increases the level of burnout [43,44], but the relationship is inverse with job dissatisfaction, concluding that job demands are not homogeneous. The identification of different types of demands with different effects is an issue discussed in the literature. Job demands are a multifaceted construct; therefore, each type of demand has a different effect [28]. Demands have a quantitative character when the professional is induced to work faster or more hours. These factors would be tight deadlines or work overload. On the other hand, the demands can be qualitative, such as role conflict and job ambiguity. Organization literature classifies the stressors on challenges and hindrances [45]. The former cause positive effects (challenges), and the latter cause negative effects (hindrances). The challenges include work overload, tight deadlines, or level of responsibility. However, the hindrances would refer to role ambiguity, role conflict, bureaucracy, or company policy issues.

Quantitative demands, such as work overload, can exert a positive influence on satisfaction, which has been called eustress (positive stress); these make the work of the internal auditor exciting and challenging [46] in performance [47]. This quality of the employee who transformed stress situations into growth challenges has been called hardiness [27]. However, the pressure caused by a lack of time can lead to dysfunctional practices [42].

Among the qualitative demands, it is observed that role conflict generates a direct and negative effect on job satisfaction and performance [46], although the level of dysfunctional practices is reduced as the task becomes more complex (role ambiguity) [42].

With regard to the audit activity, the risk of significant errors introduces a sense of fear in the members of the audit teams that can determine either a motivational effect, which stimulates a diligent practice, or a negative effect, such as the adoption of defensive strategies [34].

Hypothesis 1 attempts to prove the direct effects of the relationship between job demands and subjective well-being. The hypothesis distinguishes between the effect of quantitative and qualitative stressors.

**H1.1.**  *Quantitative demands reduce the subjective well-being of accountants.*

**H1.2.**  *Qualitative demands reduce the subjective well-being of accountants.*

### 1.2. Job Resources: Task Control and Social Support

The nature of job resources is diverse, and several meta-analysis studies reveal a motivating effect that influences work engagement [48,49]. However, the role of resources is broader since they have a dual impact. On the one hand, a high level of resources produces an extrinsic motivational effect; i.e., the individual employs an additional effort that allows the person to reduce the job demands and facilitate achievement of the objectives. On the other hand, resources generate an intrinsic motivation effect to meet the human needs of autonomy, relationship, and competition. This emotional effect also produces a positive effect on the achievement of the objectives [15,16].

The JDCS model proposes two types of resources: control over activities and social support [5,6]. Control over activities is described as having the latitude to make decisions, which consists of two constructs: autonomy in decisions and the ability to apply personal skills. Social support is a resource consisting of positive emotional exchange between employees and between these and their superiors [50]. Autonomy in decision-making increases control over activities, a factor that has proven very positive in creativity and innovation [29]. Similarly, in the professional context of this study, the internal auditors who consider that they have the capabilities to tackle their work have lower levels of burnout [51].

The processes that accountants develop contain activities structured and subjected to protocols as well as others in which the need for professional judgment becomes difficult to protocolize. The generation of routines can be positive insofar as they relate to specific tasks but cannot substitute the essentials of audit judgment. In the audit context, the audit programs of previous years are the basis for planning the job. However, the evidence on program adjustment under new audit risks is mixed, observing the lack of adjustment [52] or the change in the program under new fraud risk factors [53]. Audit tasks require different levels of professional skepticism. If the required skepticism is low, structuring programs and previous experience with the customer allows tasks to be assigned to a lower level of expert knowledge professionals, reducing the cost of auditing and releasing more experienced resources for other activities. The level of competence acquired by the accountants and the degree of autonomy in the design of procedures reduce the qualitative [54] and quantitative demands [38]. Client tenure is a resource that reduces the negative effect of work overload [55].

Social support, the organization’s trust in professionals, is a resource that produces lower levels of burnout among accountants [51,56,57].

Therefore, the literature reveals a direct and positive relationship between available resources and subjective well-being. Hypothesis 2 tests the direct relationship between resources and the construct of subjective well-being. The hypothesis distinguishes between resources related to the task control of those linked to social support.

**H2.1.**  *Task control increases the subjective well-being of accountants.*

**H2.2.**  *Social support increases the subjective well-being of accountants.*

### 1.3. The Buffer or Mediating Role of Job Resources in the Relationship between Job Demands and Subjective Well-Being

The interaction of job resources in work demands has been theoretically proposed by the JDCS model [5,6]. According to this model, the job demands can be buffered by the control that the subject has on activity and through the support that can be received from the organization itself (superiors and peers) to cope with this stress. This buffering role of job resources, which mitigates the effects of the requirement in work engagement, has been observed in several empirical papers [58,59,60].

Thus, organizational resources moderate the relationship between job demands and performance. Organizational resources include a context of organizational support and openness to innovation [30], strong leadership on time management of the teams [32], or the balance between the efforts required and granted compensation [28].

In the field of auditing firms, the support of the firm is a key resource in the management of job demands. Firms accumulate resources in the organization to manage the pressure because they trust the buffering effect, as the JDCS model predicts. Thus, accounting firms promote values and ethical behaviors that have a positive influence on the socialization of teams, which moderates role conflict [61]. Moreover, superiors have an important role in improving the performance of teams, meeting the personal needs of their members, promoting innovative ideas, properly adjusting schedule budgets [62], and promoting a culture of communication and cohesion among team members [63]. In an environment under role ambiguity, firm support buffers the relationship between task complexity and subjective well-being because professionals develop a feeling of belonging and teamwork [11].

Personal resources also exert a buffering effect; the emotional intelligence of auditors thus buffers the relationship between work overload and a measure of the quality of the auditor’s professional judgment: the level of skepticism [64].

The buffering effect of each resource type is tested with the following hypotheses:

**H3.**  *Task control buffers the relationship between job demands and the subjective well-being of accountants.*

**H4.**  *Social support buffers the relationship between job demands and the subjective well-being of accountants.*

An alternative explanation is that job demands influence resources; thus, there is an indirect effect of job demands on subjective well-being. The relationship between job demands and job resources is explored [35].

The conservation of resources theory proposes that job demands can weaken the reserves of resources to deal with other types of demands, leading to a spiral of losses. Individuals seek to build, protect, and retain their personal resources to cope with the demands of the post. When professionals are unable to achieve this, negative health effects may occur [65]. Thus, it has been observed that pressure causes team members to take a more individualistic attitude, losing the perspective of experience and reducing the performance [66], or decreasing the cognitive ability of the teams [34].

In addition, a negative relationship between job resource availability and job demands has been observed [12,13,67,68,69].

The evidence on the linear relationship of the interaction of the constructs of demand and control is not conclusive, constituting a current unresolved empirical research problem. One of the attributed causes is the existence of other factors that mediate the relationship between job demands and professional response. These factors include encouraging employees to exploit their strengths [70], personal links in the workplace [71], or the buffering effect of perceived justice in the effort–compensation link [28].

Another explanation for jobs of high status, as presented in the current investigation, is a positive relationship between job demands (such as responsibility and overload) and the accumulation of resources to deal with this increased job demand [35]. However, in jobs that are less qualified but demanding in terms of overload, a negative relationship is expected because the requirement of the position reduces the ability to accumulate resources. Therefore, this framework leads to the following hypotheses:

**H5.**  *Resources have an effect of mediation on the relationship between the quantitative demands and subjective well-being of accountants.*

**H6.**  *Resources have an effect of mediation on the relationship between the qualitative demands and the subjective well-being of the accountants.*

**Figure 1:**
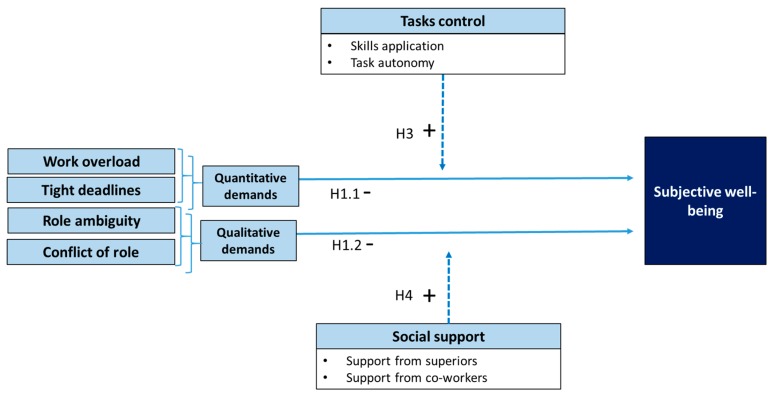
Model of the relationship between job demands, resources, and subjective well-being with a resource-moderating effect.

**Figure 2:**
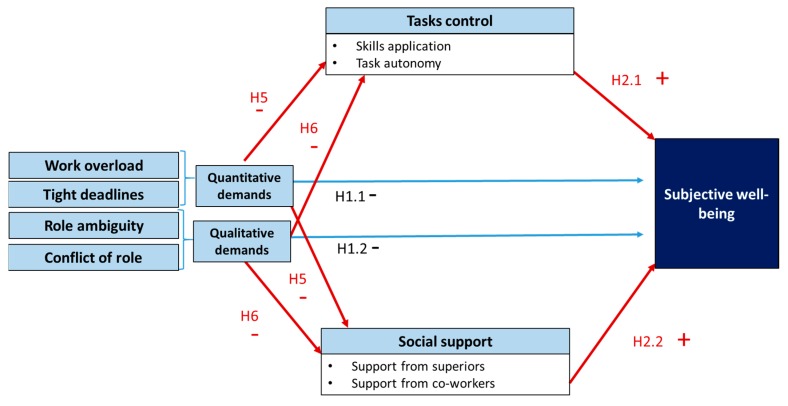
Model of the relationship between job demands, resources, and subjective well-being with the effect of mediator resources.

The models proposed are as follows (see Figure 1 and Figure 2):

Under this framework, this paper poses a double objective. First, analyze the direct effects of job demands and resources on the level of subjective well-being of accountants. Second, investigate whether resources explain or buffer the relationship between job demands and subjective well-being, i.e., whether its role is as mediator or buffering. In the business context, subjective well-being has been shown to improve the performance of workers [72,73,74,75]. Thus, the poor psychological health, e.g., burnout, has a direct negative effect on job satisfaction, increasing turnover intentions, or decreasing performance [4].

The empirical approach makes it possible to quantitatively test the raised hypotheses to respond to the research objective. However, this methodology entails some limitations that are exposed when discussing the results.

## 2. Materials and Methods

### 2.1. Sample

The sample was obtained from the sixth European working conditions survey upon request of the “accountants” codes [76]. The fieldwork was developed in 2015 with a total of 43,850 responses. The collective of accountants is formed by 739 professionals, which are integrated in codes 2411 (accountants) and 3313 (accounting associate professionals) of the International Standard Classification of Occupations.

The basic demographics of this survey are that 75.2% are women, the average age of respondents is 43.9 years, and 82.5% of the accountants carry out their activity in the private sector, with an average number of 10.9 years’ experience.

### 2.2. Measures

The dependent variable is subjective well-being, which is measured using the Well-being Index (WHO-5). This questionnaire, developed by the World Health Organization, consists of 5 items that evaluate the feelings of those surveyed during the past two weeks (e.g., “I have felt cheerful and in good spirits”) on a scale of 1 (all of the time) to 6 (at no time). The scientific community has validated this questionnaire [77], and it has displayed good internal consistency (Cronbach’s alpha: 0.84).

This research includes 4 independent variables: quantitative job demands, qualitative job demands, control over tasks, and social support mechanism. There are two types of quantitative job demands, which are measured using 3 items: one of them is work overload, which includes items such as “You have enough time to get the job done”, and the other type is tight deadlines, which includes items such as “Does your job involve working with tight deadlines?”. On the other hand, qualitative job demands are measured by an item belonging to the category of role ambiguity (“You know what is expected of you at work”). For role conflicts, the questionnaire contains two items (“Your job requires that you hide your feelings”, and “Does your main paid job involve being in situations that are emotionally disturbing for you?”).

The scale of control over the tasks integrates 4 items that measure the implementation of personal competencies (for example, “Does your main paid job involve solving unforeseen problems on your own?”) that participants must answer by yes or no. The degree of autonomy in the implementation of activities is evaluated through 7 items (for example, “Are you able to choose or change your order of tasks?” or “You have a say in the choice of your work colleagues”), which respondents must answer either with a yes or no or on a scale of 1 (always) to 5 (never).

Finally, the social support mechanism variable measures the support and guidance of superiors with 7 items (for example, “Your manager helps and supports you”). Other coworkers’ support is determined by a unique item that asks directly if “Your colleagues help and support you”, on a scale of 1 (always / strongly agree) to 5 (never / strongly disagree) for all items in the variable mechanism of social support.

Items or dimensions are integrated into the constructs in a reflective mode (mode A) or a formative mode (mode B). In the reflective mode, the observed variables (for example, items) reflect the latent variable (for example, the construct), while in the formative mode, observed variables form the latent variable.

### 2.3. Methodology

The method used to test the hypothesis is partial least squares (PLS) path modeling, a technique of structural equation modeling [78]. PLS enables the evaluation of the reliability and validity of the measures of the theoretical constructs as well as the estimate of the relationships between hypothesized constructs [79]. The software used is SmartPLS 3.2.8 to statistically test measurement and structural models [80]. Multidimensional structures are modeled using the two-stage approach [78,81].

## 3. Results

PLS models are evaluated in two stages: (i) verifying the reliability and validity of measurement models and (ii) evaluating the strength of the relationships within the structural model, as well as the explained validity of the endogenous constructs.

### 3.1. Measurement Model

The measurement model for second-order constructs and first-order dimensions shows acceptable results (see Table 1). This measurement model meets the requirements of item reliability, since all loadings of the construct subjective well-being, estimated in mode A, outweigh 0.707 [82]. This model also satisfies the requirements of construct reliability because, as shown in Table 2, the Cronbach’s alpha, Jöreskog’s rho (rho_A), and composite reliability (CR) values are higher than 0.7 [83]. Additionally, this model meets the requirements of convergent validity since the average variance extracted (AVE) is located above the critical level of 0.5 [84]. Finally, Table 2 also reveals that, according to the heterotrait–monotrait ratio (HTMT) criterion [85], the AVE meets the requirement of discriminant validity, with all the elements of the array below the threshold of 0.85 [86].

With regard to other constructs, estimated on mode B, the analysis begins testing the potential existence of multicollinearity among the items [78]. For this purpose, the variance inflation factor (VIF) should be discussed. A VIF greater than 3.3 is a sign of high multicollinearity [87]. However, multicollinearity should be considered if VIF levels exceed the critical level of 5 [80]. In this case, the maximum VIF for items and dimensions is 2.667 (see Table 1), which is below both thresholds; therefore, it can be said that multicollinearity is not concerning in this study. Finally, the magnitude and importance of the weights are examined (see Table 1). Weights provide information about how each element contributes to the dimensions and the constructs, which allows classification of the indicators according to their contribution [88].

### 3.2. Structural Model

Standard errors, statistical *t*-test, *p*-values, and corrected confidence intervals of 95% bias (BCCI: bias-corrected confidence intervals) have been generated with a technique of random resampling (5000 resamples bootstrapping) [89]. This allows us to evaluate the statistical significance of relationships set forth in the hypotheses (both direct and indirect) within the research model. Moreover, the coefficient of determination (R^2^) is the main criterion for measuring the explained variance of the endogenous constructs.

Table 3 lists the main parameters obtained for the structural model with the buffering effect evaluated in this study (structural model 1). The results show that the structural model with the buffering effect carries an acceptable predictive relevance to subjective well-being; the coefficient R^2^ is 0.223.

This model supports the direct and negative relationships between quantitative and qualitative job demands and subjective well-being variables, thus confirming H1.1 and H1.2. The model also supports the direct and positive relationship between task control and social support with the construct subjective well-being, confirming H2.1 and H2.2, respectively.

Conversely, the results do not support the buffer effect of task control and social support in the relationship between quantitative and qualitative job demands on subjective well-being, thus rejecting hypotheses H3 and H4.

Alternatively, Table 4 raises the possibility that control tasks and social support actually exercises a mediator effect on the relationship between job demands and subjective well-being (structural model 2). The results show that the structural model with a mediator effect has acceptable predictive relevance to subjective well-being, as the R^2^ amounts to 0.203. However, for mediating variables, task control and social support, the R^2^ values are 0.127 and 0.077, respectively, which means that demands do not explain most of the variance, while there are some constructs that help explain the behavior of the variable subjective well-being (Table 4).

As shown in Table 4, the structural model with a mediating effect verifies all relationships under the hypothesis of the research, both indirect and direct. First, the direct and negative relationship between quantitative and qualitative job demands and subjective well-being are ratified (H1.1 and H1.2).

Second, the structural model again confirms hypotheses H2.1 and H2.2, realizing a direct and positive significant relationship of job resources with subjective well-being with a *t*-statistic of 2.629 for control tasks and 5.586 for social support.

Third, the results show a significant negative relationship between quantitative demands with task control and social support as well as between qualitative demands and both resources. These results, as shown in Table 4, lead to the conclusion that there is empirical evidence to support the mediating effect exercised by resources on the relationship between the quantitative demands and subjective well-being (H5), as well as between qualitative and subjective well-being (H6). Both direct and indirect relations are meaningful; direct relations are more significant than indirect or mediated ones, so mediation is considered partial.

The structural model with mediating synaptic effects also supports the direct and negative relationship between quantitative and qualitative job demands and variable task control and social support (H5 and H6). Therefore, the results show that there is empirical evidence to support the former hypothesis.

## 4. Discussion

The National Institute for Occupational Safety and Health (NIOSH) focuses on the importance of the well-being of workers, their families, and communities through a series of factors relating to the work environment, such as wages, hours of work, workload and stress levels, interactions with colleagues and supervisors, and access to paid holidays [90]. More precisely, the objective of this research is to test the operation of the model JD-R in a labor context little explored at the academic level, despite the role that its protagonists (accountants) play in national economies, ensuring, among other things, transparency in financial transactions. In particular, this paper posed the challenge of evaluating the direct and indirect effects of the resources in the relationship demands and subjective well-being.

The application of structural equation models over a sample of more than seven hundred accountants allows us to reach relevant conclusions that affect the very essence of the profession.

First, the results emerge that job demands and resources have a direct effect on subjective well-being, in tune with the previous literature in the fields of organizations [17,91] and accounting. The variables linked to health worsen as job demands increase [4,43,44], while they improve as more resources are available [38,51,56,57].

The accounting profession is interesting because it is subject to stressors of a different nature, and the previous literature notes that this heterogeneity has a different well-being effect [45,46]. Moreover, this mixture that combines the demands of different natures would be the worst-case scenario for occupational health [47]. The results obtained reveal that the behavior of the job demands on the subjective well-being of the accountants takes the same sign with independence whatever the source that causes it, unlike that proposed by [45], and it can confirm the claim of [47]. This differential behavior is based on the quantitative demands that would cause an activation of the resources of the employee and give attraction to this type of job that is challenging; however, as we will discuss later, all demands consume resources of the accountant rather than activate them [46].

These results suggest that resources do not buffer the relationship between both types of job demands and the perception of subjective well-being since the buffer relationship is not significant. The evidence on interactive effects provided for in the JDCS model is scarce, and these results are in line with the findings of a review of the literature of two decades on this model [17,91]. The practical implication of this absence of an interactive effect is that in this profession, well-being comes not from increasing resources but from reducing pressure.

In contrast, resources are part of the explanation of the effect of job demands on subjective well-being. The relationship between job demands and resources is an issue of empirical verification [35]. This type of research includes empirical evidence that demonstrates that, in the accounting profession, both the quantitative and the qualitative demands consume the perception of available resources [31,66]. Consequently, the consumption of resources due to strong job demands also causes fewer resources that would have a positive effect on well-being.

As a general rule, organizations tend to make more resources available to employees when the demands of the position are more stringent; however, the results of this study show a relationship of opposite signs. This paradox could be explained from the foundations of the conservation of resources theory, which postulates that the resources of the individual apply to reduce job demands [65]. The responses indicate that the perception about the existence and value of these resources disappears because the demands are very high and, on the other hand, are more valued when the pressure is lower.

At the theoretical level, the results are consistent with the JD-R model and contribute to the mediation role of resources in the relationship between job demands and subjective well-being. The JD-R model proposes a buffering effect of the demands in the relationship between resources and motivation; in such a way that when there are many resources, greater demand plays a positive buffering effect on motivation [92]. However, the relationship of mediation that is observed in this study between professional accountants clarifies the above statement because the perception of consumption of resources to high demands would prevent that buffering role. The theoretical framework of challenges and hindrances could provide the answer because both types of demands are present in the accountancy profession, so the effect of hindrance will void a possible positive effect of challenges on professional health [47]. In this professional environment, the high job demands nullify the positive effect of challenges (quantitative job demands), in line with the arousal theory. This theory describes an inverted U-shaped relationship between demands and well-being that has received broad support in the literature [28,29,30,31,32,33,34].

These results have important practical implications because they highlight that unless a certain level of pressure is exceeded, accounting experts have more resources related to task control and/or social support and are not going to improve subjective well-being. Only pressure reduction can produce important personal benefits to workers, directly and indirectly, through the role played by job resources.

Nonetheless, cross-sectional research lacks causality quality. This limitation could be overcome with a longitudinal design, but this is not possible using the public and recognized sample that has been employed. In addition, measures on the research come from a self-perception questionnaire in which the answers are subjective perceptions and not objective states.

This research opens new questions about the role of work–life balance on the job demands and subjective well-being relationship. Thus, work–life balance could be a resource that buffers the job demands effect on well-being or could be an additional stressor for the accountant. A second research question is based on the effect of culture or religious feeling on managing job demands and its effect on subjective well-being. A third future line of research is to analyze whether the practical implication of this paper is equally effective for different categories in accountancy firms. Different levels in a firm assume different types of demands and have access to dissimilar job resources.

## 5. Conclusions

The aim of this paper was twofold. First, this research assesses the influence of job demands and job resources on accountants’ subjective well-being and second, the model analyzes the buffered or mediated role of job resources in the link among job demands and accountants’ subjective well-being. A scarce subjective well-being has been negatively affected by health, job satisfaction, performance, and turnovers intentions.

The accounting profession brings together diverse human nature demands that cause negative effects on subjective well-being. Conversely, job resources have a positive effect on subjective well-being. The indication of the mediation of resources on the relationship between subjective well-being and demands shows that, at the highest demand levels, professionals perceive that they have fewer resources against greater demands. This finding has theoretical and practical implications of interest.

Theoretically, the JDCS model postulates resources to moderate the relationship between demands and strain. The results do not confirm this interactive relationship, but they manifest a mediating role of resources given that there is an indirect relationship of the demands on subjective well-being through resources, which can be a perception of the accountant. From a practice perspective, these results are quite relevant because they affirm that the subjective well-being of accounting professionals can improve more efficiently, reducing the pressure instead of increasing the job resources available to the professionals.

## Figures and Tables

**Table 1:** Mediation model of measurement.

Variable		Loads		Weights		VIF ^1^
**1**	**Quantitative demands**			**n/a**		**n/a**
	Your job involves working at very high speed			0.067		1.600
	Your job involves working with tight deadlines			0.054		1.571
	Enough time to get the job done			0.964	**	1.086
**2**	**Qualitative demands**			**n/a**		**n/a**
	Emotionally disturbing situations			0.432	**	1.061
	You know what is expected of you at work			0.854	**	1.010
	Your work requires you to hide your feelings			0.041		1.052
**3.1**	**Application of personal skills**			**0.186**		**1.014**
	Short repetitive tasks of less than 10 minutes			0.234		1.044
	Solving unforeseen problems on your own			0.419		1.150
	Monotonous tasks			0.889		1.058
	Learning new things			−0.027		1.149
**3.2**	**Autonomy in planning activities**			**0.961**	**	**1.014**
	Ability to choose your order of tasks			0.045		1.426
	Ability to choose your methods of work			−0.120		1.469
	Ability to choose your speed or rate of work			0.435		1.327
	You are consulted before objectives are set for your work			0.175		1.373
	You have a say in the choice of your work colleagues			−0.081		1.291
	You are able to apply your own ideas in your work			0.543	**	1.545
	You can influence decisions that are important to your work			0.334	*	1.712
**4.1**	**Support and guidance from superiors**			**0.871**	**	**1.097**
	Your manager helps and supports you			0.131		1.457
	Your boss respects you as a person			0.530	**	1.748
	Your boss gives you recognition			0.293		1.897
	Your boss is successful in getting people to work together			0.004		2.123
	Your boss is helpful in getting the job done			−0.065		1.779
	Your boss provides useful feedback			0.192		2.047
	Your boss encourages and supports your development			0.148		2.667
**4.2**	**Support from other colleagues**			**0.297**		**1.097**
	Your colleagues help and support you			1.000	**	1.000
**5**	**Subjective well-being**	**n/a**				
	I have felt cheerful and in a good mood	0.848	**			
	I have felt calm and relaxed	0.857	**			
	I have felt active and vigorous	0.857	**			
	I woke up feeling fresh and rested	0.844	**			
	My daily life has been filled with things that interest me	0.778	**			

The significance of loads and weights has been estimated by a bootstrapping 95% confidence interval (n × 5000 samples). * *p* < 0.01; ** *p* < 0.001 (based on t (4999), two-tail test). ^1^ Variance inflation factor (VIF) is used as an indicator of multicollinearity.v.

**Table 2:** Construct Reliability. Convergent validity and discriminant validity.

Variables	Cronbach’s Alpha ^1^	rho_A ^1^	Composite Reliability ^1^	Average Variance Extracted (AVE) ^2^			
Subjective well-being	0.893	0.894	0.921	0.701			
**Discriminant validity**							
***Heterotrait–Monotrait Ratio HTMT*** ^3^							
	Skills application	Co-workers support	Superiors support	Task autonomy	Subjective well-being	Qualitative demands	Quantitative demands
Skills application							
Co-workers support	0.191						
Superiors support	0.130	0.501					
Task autonomy	0.369	0.266	0.460				
Subjective well-being	0.174	0.168	0.345	0.277			
Qualitative demands	0.498	0.327	0.389	0.520	0.491		
Quantitative demands	0.388	0.131	0.241	0.227	0.366	0.622	

^1^ Cronbach’s alpha, Jöreskog’s rho (rho_A), and composite reliability (CR) assess construct reliability. ^2^ Average Variance Extracted (AVE) assess convergent validity. ^3^ Heterotrait–monotrait ratio of correlations (HTMT) is an approach based on the multitrait–multimethod matrix to assess discriminant validity.

**Table 3:** Structural Model 1 (Buffer effect).

R^2^ Subjective Well-Being: 0.223 Relationship	Original Sample (O)	T Statistics	*P* Value		2.5%	97.5%	Significative
***Direct effects***							
Quantitative demands -> Subjective well-being	−0.221	5.695	0.000	***	−0.292	−0.141	Sig.
Qualitative demands -> Subjective well-being	−0.098	2.494	0.013	*	−0.170	−0.015	Sig.
Task control -> Subjective well-being	0.123	3.284	0.001	***	0.049	0.194	Sig.
Social support -> Subjective well-being	0.248	6.073	0.000	***	0.166	0.325	Sig.
Task control*Qualitative demands. -> Subjective well-being	0.020	0.429	0.668		−0.077	0.110	No Sign.
Task control*Quantitative demands. -> Subjective well-being	−0.002	0.050	0.960		−0.095	0.092	No Sign.
Social support*Qualitative D. -> Subjective well-being	0.025	0.645	0.519		−0.055	0.097	No Sign.
Social support*Quantitative D. -> Subjective well-being	−0.047	1.135	0.257		−0.129	0.032	No Sign.

Bootstrapping 95% confidence intervals bias corrected (*n* = 5000 subsamples). *** *p* < 0.001; ** *p* < 0.01; * *p* < 0.05 [based on *t* (4999), two-tailed test].

**Table 4:** Structural Model 2 (Mediating effect).

*R*^2^ Subjective Well-Being: 0.203; *R*^2^ Task Control: 0.116; *R*^2^ Social Support: 0.077 Relationship	Original Sample (O)	T Statistics	*P* Value		2.5%	97.5%	Significative
**Direct Effects**							
Quantitative demands -> Subjective well-being	−0.269	6.057	0.000	***	−0.349	−0.171	Sig.
Quantitative demands -> Task control	−0.193	5.183	0.000	***	−0.263	−0.116	Sig.
Quantitative demands -> Social support	−0.179	4.003	0.000	***	−0.265	−0.090	Sig.
Qualitative demands -> Subjective well-being	−0.157	3.570	0.000	***	−0.236	−0.062	Sig.
Qualitative demands -> Task control	−0.223	5.870	0.000	***	−0.291	−0.146	Sig.
Qualitative demands -> Social support	−0.165	3.298	0.001	***	−0.258	−0.058	Sig.
Task control -> Subjective well-being	0.108	2.629	0.009	**	0.026	0.188	Sig.
Social support -> Subjective well-being	0.239	5.586	0.000	***	0.156	0.322	Sig.
***Indirect Effects***							
Quantitative demands -> Subjective demands	−0.064	4.002	0.000	***	−0.098	−0.036	Sig.
Qualitative demands -> Subjective demands	−0.064	3.748	0.000	***	−0.098	−0.033	Sig.

Bootstrapping 95% confidence intervals bias corrected (*n* = 5000 subsamples). *** *p* < 0.001; ** *p* < 0.01; * *p* < 0.05 [based on *t* (4999), two-tailed test].
